# Editorial: Health and welfare problems of farm animals: prevalence, risk factors, consequences and possible prevention solutions

**DOI:** 10.3389/fvets.2023.1238852

**Published:** 2023-07-04

**Authors:** Nikola Čobanović, Luisa Magrin

**Affiliations:** ^1^Department of Food Hygiene and Technology, University of Belgrade, Belgrade, Serbia; ^2^Department of Animal Medicine, Production and Health, University of Padua, Padua, Italy

**Keywords:** Artificial Intelligence, calf welfare, data, farmer perceptions, lameness, muscularity and body condition score, thermography, qualitative behavioral assessment

According to the forecasts, the global population is expected to grow by two billion by 2050 and, subsequently, the demand for animal products especially those obtained from animal welfare-friendly production systems to satisfy high consumer requirements (1–4). However, the vast literature points out that intensive farming systems aimed at maximizing productivity per animal generate negative impacts on the health and welfare of farm animals, such as increased emotional stress (5, 6), risk of injuries, and physiological and anatomical disorders (*i.e.*, higher prevalence of lameness, etc.) (7), and reduced life expectancy (8, 9). There are many causes often linked to the nutritional and management practices and housing regimes adopted by the farmers (10, 11). Improving the health and welfare of farm animals can enhance their growth rate and reproduction, the quantity and quality of the final marketed product, and, as a consequence, the economic efficiency of the farms (12). Moreover, it may offer significant benefits for human health in the long term, contributing to a reduction in antibiotic use at non-therapeutic levels for growth promotion or disease prevention or in the use of some contaminants (i.e., pesticides) on crops to feed farm animals (13, 14). Despite increasing interest in this research field in the past several decades, the prevalence and consequences of health and welfare problems in intensive farming systems are alarming, and thus, there are still many concerns to be dealt with. Effective preventive and corrective procedures or protocols and new diagnostic methods to be implemented to identify animal welfare risks are crucial in ensuring animal and human health.

This Research Topic consists of a collection of nine studies, two on pigs and seven on dairy cows, that deal with some of the current health challenges for farm animals and with alternative approaches to assessing their welfare. Concerning the existing problems being experienced by farmers, lameness is still one of the most impactful issues regarding animal welfare and economic losses for cattle (15, 16). The potential effects of lameness on animal behavior and a viable treatment protocol for its recovery are some of the topics discussed in two studies of the collection (Gündel et al.; Sadiq et al.). Gündel et al. reported that Jersey cows could behave differently to lameness compared to other breeds and that feeding indicators might not be a useful tool for early detection of lameness. To obtain better recovery rates, treatment protocol consisting of therapeutic trim, hoof block, and pain management, in combination with early detection of cow lameness, was suggested by Sadiq et al.. Among the new alternatives to assess animal welfare, Rosengart et al. reported that thermography, coupled with Artificial Intelligence systems, could be a promising diagnostic tool for detecting diseased sows and piglets at the earliest time. In addition, Lutz et al. explored the accuracy of a quick and cost-effective data-based prediction of dairy cow welfare status. The authors demonstrated that data-based parameters have only potential to provide useful information on specific welfare aspects rather than to provide a comprehensive predictive tool for dairy welfare status at the herd level. In the study reported by Nadlučnik et al., differences between farmers' perceptions and real pig welfare conditions were evaluated. Despite the fact that farmers are aware of animal welfare importance, they follow only minimal statutory requirements, indicating that there is considerable room for improvement, especially regarding biosecurity on pig farms. Another topic covered in this Research Topic concerns the importance of the role of some environmental or resource-related actions as preventive measures to reduce animal stress. Specifically, two detailed systematic reviews reported the best feeding and social management (housing) practices for improving the welfare of pre-weaned calves [Carulla et al. (a); Carulla et al. (b)]. The authors reported that the most important gaps in knowledge regarding dairy calves are the lack of a clear protocol for administering milk replacers to reduce hunger and the best management of weaning to reduce stress, as well as the information regarding optimal time to separate the calf from its mother. One study investigated using the qualitative behavioral assessment whether the provision of different forms of environmental enrichment resources would impact the affective states of housed dairy cows (Russell et al.). The results obtained in this study demonstrated that simple modification to the housed environment, access to a novel object and outdoor space positively influenced the affective lives of commercially housed dairy cows. Lastly, this Research Topic also contains an innovative study, authored by Buonaiuto et al., who provided new predictive indicators (muscularity and body condition score) of the stayability and longevity in a dual-purpose cattle population.

## Author contributions

All authors listed have made a substantial, direct, and intellectual contribution to the work and approved it for publication.
